# Carbon-Based Functional Nanomaterials: Preparation, Properties and Applications

**DOI:** 10.3390/ma19030537

**Published:** 2026-01-29

**Authors:** Valery N. Khabashesku

**Affiliations:** Department of Materials Science and Nanoengineering, Rice University, Houston, TX 77005, USA; khval@rice.edu

The age of carbon nanomaterials was jump-started by the remarkable discovery of buckminsterfullerene C_60_ in 1985 by Smalley, Kroto, and Curl [1]. This landmark work was further advanced by the synthesis of carbon nanotubes and other nanostructured modifications of carbon, such as carbon nanofibers, nanodiamonds, graphene, and carbon dots, and the theoretical prediction of over a hundred more nanoscale carbon allotropes. These allotropes are formed due to the ability of carbon to exist in several hybridization states, sp, sp^2^, and sp^3^, which prompt self-assembling of carbon into zero-, one-, two-, and three-dimensional nanostructures. Properties of nanomaterials are size-dependent in the dimension scale of 1–100 nm due to “quantum effects” and increased surface area. As a result, the properties of the particle, including its melting point, fluorescence, electrical conductivity, magnetic permeability, and chemical reactivity, adjust in accordance with its size, thereby facilitating the development of innovative applications [2]. Additionally, carbon nanomaterials can be functionalized to obtain desired surface properties to either alter the surface wettability or to allow incorporation into different matrices [3]. This helps to expand their functional properties and applications in diverse areas, starting from biology, medicine, and electronics, and even reaching out to aerospace [4] and oil and gas industries [5]. The advances in the scale-up synthesis of different carbon nanomaterials made them commercially available at a reasonable cost and facilitated extensive applied research not only in academia but also in the industry. The scope of this Special Issue is to illuminate the most recent developments of research on the production, characterization, properties, and broad applications of multifunctional carbon-based nanomaterials, as well as to cover the current challenges and opportunities in their industrial acceptance and potential technological scale-up. This Editorial mainly provides a summary of the eight research articles included in this Special Issue.

Starting from their discovery by Iijima [6,7], carbon nanotubes (CNTs), representing a one-dimensional (1D) carbon nanostructure built from sp^2^-state carbon atoms, have been a subject of extensive research. The unique combination of chemical, mechanical, and electrical properties, as well as outstanding UV-blocking and oxidation resistance abilities of CNTs, became of particular interest to the textile industry [8]. Due to their remarkable colloidal stability in water, two guanidinylated derivatives of hyperbranched polyethyleneimine (GPEI5k and PEI 25K) functionalized multi-walled oxidized CNTs (oxCNTs@GPEI5K and oxCNTs@GPEI5K) were selected for evaluation of antibacterial activity on Gram (−) *Escherichia coli* and Gram (+) *Staphylococcus aureus* bacteria in work [9]. The derivative oxCNTs@GPEI5K, found to be most promising, has subsequently been used as a finishing agent for wool fabric. As a result, the GPEI-functionalized oxCNTs derivative due to uniform distribution and good adhesion to the wool yielded wool fabrics with sustained antibacterial properties even after multiple washing cycles. As an additional benefit, these textiles also demonstrated enhanced UV protection, highlighting the potential of CNTs for multifunctional applications in wearable textiles.

A peculiar example of carbon materials’ function has been demonstrated on the example of carbon fiber paper (CFP), possessing a combination of air permeability, light surface density, and suitable flexibility. Due to these properties, CFP was used as a wearable material for applications in the field of flexible electromagnetic shielding (ES). The technique that was previously developed [10] to prepare CFP with a gradual thickness was employed in the study [11]. The ES efficiency of the CFP was found to vary with the frame-like thickness pattern distribution. It was noted that at a 5 mm thickness, the CFP shows a remarkable overall ES performance, reaching 63.46 dB, thus confirming that CFP can be utilized in the future as a prospective ES material.

Carbon nanostructures, according to article [12], can participate in energy transportation and conversion during tribological processes. Tribosystem, comprising a lubricating oil, carbon nanomaterials, and zinc dialkyldithiophosphate (ZDDP) additive combined with antistatic additive (ASA) for jet fuels, has been evaluated in contact with the metal. It was found that carbon-based nanostructures, such as CNTs, AuCNTs (carbon nanotubes surface-coated with Au), graphene, and fullerene C_60_, affect the kinetics of chemical reactions of ZDDP during tribological processes. CNTs, in particular, were shown to act as catalysts in tribochemical reactions of ZDDP, while graphene and fullerenes demonstrated only a minor effect. In comparison, AuCNT alone behaves as an inhibitor during ZDDP’s triboreaction, while ASA, in cooperation with CNT and AuCNT, due to discharging electric charge/energy, notably slows down the same reaction.

Tribological properties of hydrogenated amorphous carbon (a-C:H) and amorphous carbon/gold (a-C/Au) composite films were studied and compared at different humidities [13]. This study was motivated by the fact that although amorphous carbon is an excellent lubricating material, its tribological properties can be substantially influenced by humidity [14]. The friction coefficients (FC) of a-C:H and a-C/Au films were tested using a rotational ball-on-disk tribometer. Spectroscopy and HRTEM analysis of the morphology and structure of the sliding interface and first-principle calculations of the adsorption energy of water molecules on different surfaces, including steel, were performed. According to the obtained results, the FC of the a-C:H film and the area of transfer film increase with rising humidity, which can be explained by the water molecule enhancement effect of the interaction between the a-C:H film and the steel surface. On the contrary, the friction coefficient values of the a-C/Au film demonstrate low sensitivity to humidity since the formed Au transfer film interacts weakly with water molecules. These findings help develop a promising strategy for emerging environment-adaptive amorphous carbon films and intelligent lubricating films.

The research presented in article [15] focused on one of the problems in the coal industry, that is, the presence of quinoline-insoluble (QI) material in coal tar and coal tar pitch, which adversely influences the properties of subsequent carbon materials and composites [16]. The QI material is mainly composed of pyrolytic carbon, pitch coke, and tar solids, whereas the primary QI content is derived from the nanoparticles formed by decomposition of hydrocarbons. In this work, Lewis acid catalysts, AlCl_3_ and CuCl_2_, enabled a polycondensation process to cause heterogeneous nucleation of QI particles in heavy coal tar. As a result, mesocarbon microbeads (MCMBs) were produced, providing a much easier way to filter them off and remove them from the QI content of coal tar pitch. The developed method can also provide working ideas for the purification and preparation of high-quality carbon materials.

Article [17] describes the production of carbon-based highly porous bioadsorbents from household waste, commonly generated in immense quantities. The examined organic waste consisted of heavily roasted coffee residue, regular roasted coffee residue, potato peelings, tea residue, green walnut shells, walnut shells, and green coffee residue. A two-stage modification process consisting of 5-step carbonization (I stage) and 6-step activation with NaOH solution (II stage) has been developed for the waste. Based on physico-chemical analysis of each sample at each processing stage, it was found that the bioadsorbents derived from roasted coffee residue with a specific surface area of 1580 m^2^/g and a pore volume of 0.84 cm^2^/g, and potato peels with a surface area of 1604 m^2^/g and a pore volume of 0.65 cm^2^/g, are the most effective. This research is important for the selection of the best organic waste as a raw material for the commercial production of bioadsorbents applied in environmental protection under the strategy of waste management and sustainable development.

Flexible sensors (FS) based on laser-induced graphene (LIG) became of extensive use in smart healthcare, smart skin, and wearable devices as seen in the past decade [18]. The LIG technology-based fabrication of FS and their applications in human–computer interaction (HCI) systems have been demonstrated in article [19]. LIG was generated in the polyimide film by direct laser irradiation under ambient conditions using a CO_2_ laser [20] and then used for the fabrication of FS. When bent over, such LIG-based sensors showed a response time of 160 ms and a recovery time of 140 ms. Also, the FS showed exceptional durability. Even after 1000 cycles of bending, the FS structure remained intact. The integration of the FS and a flexible glove was used as a prototype of an HCI system for application demonstration. Such an HCI system could detect the bending motions of different fingers and translate these motions into computer mouse movements on screen. It is anticipated that such a newly designed LIG-based flexible HCI system can assist people with limited mobility to control a virtual keyboard or mouse pointer.

A two-dimensional (2D) layered carbide/nitride structure, called MXenes [21], possesses high metallic conductivity and pseudocapacitance and is particularly suitable for printing applications due to its good hydrophilicity. However, aqueous dispersions of MXenes are prone to oxidation; therefore, enhancement of their oxidation stability is critical. The paper [22] reported that the introduction of sodium L-ascorbate as the antioxidant stabilizes MXene aqueous dispersions for the long term. Such stabilized MXene dispersions upheld steady electrochemical properties. The supercapacitor made with antioxidant-added MXene as the electrode, even after 60 days of storage, demonstrated an excellent specific capacitance of 381.1 F/g at a scan rate of 5 mV/s and a good retention rate of 92.6% after 10,000 consecutive cyclic voltammetry measurements. These results demonstrate an efficient and low-cost approach to utilizing MXene aqueous dispersions for large-scale applications in energy storage systems.

Compared to the other 1D nanostructured carbon materials, such as single- and multi-walled CNTs, a double-walled carbon nanotube (DWNT) is composed of two concentric graphene cylinders and exhibits superior mechanical strength and electrical and thermal conductivity. These nanotubes exhibit a tensile strength that is more than 20 times that of copper despite being a 1/6 fraction of its weight. Additionally, DWNTs have a current-carrying capacity 1000 times greater than copper, making them a far more efficient conductor than typical electrical wiring. These remarkable properties make them an ideal replacement for copper wiring—potentially revolutionizing many sectors, including the electronics, telecommunications, aerospace, and oil and gas industries—and enable extensive research on methods for the fabrication of DWNT into fibers and electric wires [23,24]. Two major types of processes are currently being developed: (i) wet spinning of high-concentration DWNTs dispersed in an appropriate solvent with the added surfactants or solubilized in acid/superacid media [25] and (ii) direct spinning of DWNTs continuously grown in a vertical CVD reactor by a high-temperature catalytic process [26,27]. Wet-spinning methods, however, have limitations in terms of large-scale production and roll-to-roll manufacturing. The method only works if nanotubes can be readily dispersed in solution, which can only be achieved through the use of surfactants and short carbon nanotubes. Such requirements prevent the utilization of long, high-purity nanotubes in the spinning process—properties that are crucial in the synthesis of highly conductive nanotube fibers. In contrast, the continuous process for direct spinning of DWNT fibers from the CVD reactor does not have such limitations. The method utilizes the down-flow vertical tube reactor equipped with a programmable multi-zone high-temperature furnace where carrier gases and reagents are fed through the reactor top flange. Full-length DWNTs are grown at a high production rate on catalyst particles generated in situ to provide a fine nanotube aerosol, which is directly spun into thin fibers composed of well-aligned nanotubes. Followed by doping and densification processing steps, this method already produced DWNT wires with the electric conductivity (resistance—7 × 10^−6^ ohm·cm) becoming close to Cu (1.7 × 10^−6^ ohm·cm) and as long as 5 km [28].

In summary, the articles comprised in this Special Issue showcase the functional potential and diverse methods of preparation of carbon nanomaterials in a diverse array of applications, including wearable textiles with antibacterial and UV-shielding properties, electromagnetic shielding materials, flexible sensors for human–computer interaction, energy storage systems, coal purification, and organic waste management. The key factors affecting most of these applications are the conditions of preparation and chemical functionalization of carbon nanomaterials to modify their surface properties and improve their solubility. Future research in this field is expected to address the growing need for atomic and molecular precision level of control in the preparation of carbon nanostructures and their surface functionalization when specific material properties are well-defined by structure–property relationships [29]. This is particularly important both for a deeper fundamental understanding of how carbon and other nanomaterials behave under different conditions and for advancing a variety of applications that require reproducible and high-performance materials. Narrowing the knowledge gap in this area will help in justified decision-making on the feasibility of further commercialization of carbon-based nanomaterials.
